# A Capsid Virus-Like Particle-Based SARS-CoV-2 Vaccine Induces High Levels of Antibodies and Protects Rhesus Macaques

**DOI:** 10.3389/fimmu.2022.857440

**Published:** 2022-04-05

**Authors:** Ariane Volkmann, Gerrit Koopman, Petra Mooij, Ernst J. Verschoor, Babs E. Verstrepen, Willy M. J. M. Bogers, Manja Idorn, Søren R. Paludan, Søren Vang, Morten A. Nielsen, Adam F. Sander, Carolin Schmittwolf, Hubertus Hochrein, Paul Chaplin

**Affiliations:** ^1^ Bavarian Nordic GmbH, Martinsried, Germany; ^2^ Department of Virology, Biomedical Primate Research Centre (BPRC), Rijswijk, Netherlands; ^3^ Department of Biomedicine, Aarhus University, Aarhus, Denmark; ^4^ Department of Molecular Medicine, Aarhus University Hospital, Aarhus, Denmark; ^5^ Centre for Medical Parasitology at Department for Immunology and Microbiology, Faculty of Health and Medical Sciences, University of Copenhagen, Copenhagen, Denmark; ^6^ Department of Infectious Disease, Copenhagen University Hospital, Copenhagen, Denmark; ^7^ AdaptVac Aps, Hørsholm, Denmark; ^8^ Bavarian Nordic A/S, Hellerup, Denmark

**Keywords:** virus-like particles (VLP), SARS-CoV-2, corona, COVID-19, broad neutralizing antibodies, protection, non-human primates (NHP), variants of concern (VOCs)

## Abstract

Severe acute respiratory syndrome coronavirus 2 (SARS-CoV-2) has caused a worldwide pandemic. Here, we present non-human primate immunogenicity and protective efficacy data generated with the capsid virus-like particle (cVLP)-based vaccine ABNCoV2 that has previously demonstrated immunogenicity in mice. In rhesus macaques, a single vaccination with either 15 or 100 μg ABNCoV2 induced binding and neutralizing antibodies in a dose-dependent manner, at levels comparable to those measured in human convalescents. A second vaccine administration led to a >50-fold increase in neutralizing antibodies, with 2-log higher mean levels in the 100-μg ABNCoV2 group compared with convalescent samples. Upon SARS-CoV-2 challenge, a significant reduction in viral load was observed for both vaccine groups relative to the challenge control group, with no evidence of enhanced disease. Remarkably, neutralizing antibody titers against an original SARS-CoV-2 isolate and against variants of concern were comparable, indicating a potential for broad protection afforded by ABNCoV2, which is currently in clinical testing.

## Introduction

Coronavirus disease 2019 (COVID-19), caused by severe acute respiratory syndrome coronavirus 2 (SARS-CoV-2), has been classified as a pandemic by the World Health Organization in March 2020. The disease is characterized by fever and respiratory problems. Although the majority of the infections cause relatively mild disease, approximately 20% of the patients develop severe acute respiratory syndrome that requires hospitalization, and approximately 2% succumb to the infection (1).

Given the substantial mortality and morbidity caused by this virus, there is an urgent need for effective vaccines for the general population. Several vaccines have been approved, including mRNA vaccines, adenovirus vector-based vaccines, and inactivated virus vaccines, which in some cases, such as mRNA vaccines, afford high protection rates of >90%. However, insufficient doses are available for global vaccination and a drop in efficacy against some newly emerging variants of concern has been noted (2–4). New vaccine candidates that are currently being developed need to be evaluated in suitable animal models for safety, immunogenicity, and efficacy in order to select the best candidates for further clinical development. Recently, the immunogenicity of ABNCoV2, a cVLP-based vaccine displaying the receptor-binding domain (RBD) of the SARS-CoV-2 spike protein, has been described (5). Mice vaccinated with the vaccine formulated in a squalene-in-water adjuvant developed high levels of serum anti-spike antibodies and virus neutralization antibody titers comparable to those found in serum from patients that had recovered from COVID-19. Following booster vaccination, the neutralization titers in mice exceeded those measured after natural infection at serum dilutions above 1:10,000. The induction of IgG2a and IgG2b isotypes, as well as IFN-γ producing CD4 T cells, all characteristic of a Th1 response, suggested a reduced risk for potential vaccine-related enhanced disease. Due to the nanoparticle size and repetitive display of antigens, VLPs are highly immunogenic and are already used as successful prophylactic vaccines in other indications, e.g., against human papillomavirus, hepatitis B virus, and hepatitis E virus (6).

The cVLP vaccine ABNCoV2 presented here was tested at two different doses, using two immunizations of non-human primates (NHP) within a 14-week interval. The long interval of 14 weeks was chosen to be able to study the longevity of the immune response after vaccination. Six weeks after the second immunization, efficacy against infection was tested by experimental challenge with SARS-CoV-2. In contrast to previous studies in mice (5), ABNCoV2 was not adjuvanted in order to explore the efficacy of a SARS-CoV-2 vaccine formulation with reduced reactogenicity and to avoid potential shortages of adjuvant for global vaccinations.

## Material and Methods

### Animals

The study was performed on 12 adult male and four female rhesus macaques (*Macaca mulatta*). Males were divided into two vaccine groups and one non-vaccinated challenge control group (four males each), and two females each were added to the two vaccine groups (**Supplementary Table 1**) to evaluate the effect of the vaccine in males and females. No obvious effect of sex on vaccine immunogenicity and efficacy was seen. The animals underwent a full physical examination prior to entering the study. They were negative for antibodies to simian T-cell leukemia virus (STLV) and simian retrovirus (SRV) and negative for binding antibodies to the RBD of the spike protein of SARS-CoV-2. The study was reviewed and approved by the Dutch “Centrale Commissie Dierproeven” (AVD5020020209404-2) according to Dutch law, article 10a of the “Wet op de Dierproeven,” and BPRC’s Animal Welfare Body (IvD).

At the time of vaccination, NHP were 4 to 8 years old and weighed 5.5 to 11.7 kg (**Supplementary Table 1**).

### Vaccine and Vaccinations

ABNCoV2 (previously named RBDn-CLP) was generated as previously described (5). Briefly, a split-protein Tag/Catcher system (7–9) was used to conjugate and display the Wuhan RBD antigen aa319-591 (Sequence ID: QIA20044.1) on the protein surface of preassembled bacteriophage AP205 cVLPs (5). For vaccinations, 15 or 100 μg ABNCoV2 in 0.4 ml PBS with 200 mM sucrose was administered twice intramuscularly in the upper left leg, 14 weeks apart.

### SARS-CoV-2 Challenge

Six weeks after the second immunization, all 12 vaccinated animals and four non-vaccinated challenge control animals were exposed to 10^6^ TCID_50_ SARS-CoV-2 *via* the combined intranasal (0.25 ml/nostril) and intratracheal (4.5 ml) routes using the CoV isolate BetaCoV/German/BavPat1/2020 (European Virus Archive, Germany) and monitored for 2 weeks.

### Quantification of Subgenomic mRNA

Tracheal and nasal swabs, as well as bronchoalveolar lavage (BAL) samples, were analyzed for the presence of subgenomic mRNA (sgRNA) of coronavirus using a quantitative real-time PCR as previously described (10). Viral RNA was isolated using a QIAamp Viral RNA Mini kit (Qiagen Benelux BV, Venlo, The Netherlands) following the manufacturer’s instructions. The RT-PCR assay was carried out using the Brilliant II QRT-PCR Core Reagent Kit, 1-Step kit (Agilent Technologies BV, Amstelveen, The Netherlands), according to the instructions provided by the manufacturer in a 25-μl volume with final concentrations of 600 nM for both primers, 200 nM for the probe, and 5 nM MgCl_2_, using 10 μl RNA, extracted from 140 μl sample volume. RNA was reverse transcribed for 30 min at 50°C. Then, after a 10-min incubation step at 95°C, the cDNA was amplified for 45 cycles, consisting of 30 s denaturation at 95°C, followed by a 1-min annealing extension step at 60°C. All the reactions were carried out with an iQ5 Multicolor Real-Time PCR Detection System (Bio-Rad Laboratories BV, Veenendaal, The Netherlands).

### Cytokines and Chemokines

Serum samples and BAL fluid were tested for the presence of cytokines and chemokines CXCL9, CXCL10, CXCL11, CCL2, CCL3, CCL4, CCL5, CCL11, IL-1β, IL-6, IL-8, IFN-γ, and TNF-α using the LEGENDplex assay (Biolegend LEGENDplex™ NHP Chemokine/Cytokine Panel, 13-plex, art. nr. 740317) according to the manufacturer’s manual. Samples were measured on an Aurora machine (Cytek, Fremont, CA, USA) and analyzed by using company software.

### Enzyme-Linked Immunosorbent Assay

Preimmunization serum samples, as well as serum samples obtained at weeks 2, 4, 12, 16, 18, 20, and 22 (2 weeks after SARS-CoV-2 challenge), were tested for the presence of anti-SARS-CoV-2 spike protein RBD IgG using an enzyme-linked immunosorbent assay (ELISA) with Wuhan-RBD coating antigen (ExpreS^2^ion Biotechnologies, Agern Allé 1, DK-2970 Horsholm, Denmark). For comparison, 10 human plasma samples positive for SARS-CoV-2 antibodies (SeraCare, Performance Panel 0820-0410) were included in the assay. The RBD-specific IgG levels were calculated against a standard curve made with a monoclonal antibody specifically directed against the RBD of the spike protein of SARS-CoV-2 that was used in a concentration range of 500–78,125 ng/ml. Samples were run in eight different dilutions. Only the OD values that fell within the linear range of the curve were used for calculating the antibody concentration. All samples and standards were run in duplicates and measurements were discarded if CV values of the duplicates were above 25%.

### Plaque Reduction Neutralization Test

SARS-CoV-2 neutralizing antibodies in serum samples obtained at weeks 2, 4, 12, 16, 20, and 22 (2 weeks after virus inoculation) were measured by live virus plaque reduction neutralization test (PRNT) as previously described (5). For comparison, 10 human plasma samples positive for SARS-CoV-2 antibodies (SeraCare, Performance Panel 0820-0410) were included in the assay. PRNT50 titers were calculated as IC50 using a 4PL non-linear fit model of the software GraphPad Prism 9.0.2. The Wuhan-like, early European B.1. isolate (Freiburg, FR-4286, GISAID accession no. EPI_ISL_852748) was kindly provided by Professor Georg Kochs, University of Freiburg. The B.1. lineage differs from Wuhan (WA1/2020) by the two amino-acid substitutions aa:S:D614G and aa:Orf1b:P314L only, which were acquired early after SARS-CoV-2 emergence in Europe and are present in subsequent B.1 lineage variants, including the alpha, beta, and delta variants.

SARS-CoV-2 variant neutralization was assessed using the same PRNT as described (5), using serum samples obtained 2 weeks after the second vaccination. For these assays, the SARS-CoV-2 variant of concern alpha (B.1.1.7, NCBI GenBank accession no. MZ314997) was kindly provided by Professor Arvind Patel, University of Glasgow, United Kingdom; beta (B.1.351, GISAID accession no. EPI_ISL_678615) was kindly provided by Professor Alex Sigal, African Health Research Institute, South Africa; and delta (B.1.617.2, SARS-CoV-2/DK/SSI-H11, NCBI GenBank accession no. OM444216) was kindly provided by Statens Serum Institute, Denmark. SARS-CoV2 B.1, B.1.1.7, and B.1.617.2 were propagated in VeroE6 cells expressing human TMPRSS2 (VeroE6-hTMPRSS2; kindly provided by Professor Stefan Pöhlmann, University of Göttingen) by infection with MOI 0.05. SARS-CoV2 B.1.351 was propagated in human A549 cells expressing human ACE-2. A supernatant containing new virus progeny was harvested 72 h post-infection. The supernatant was first centrifuged for 10 min at 3,000×*g* to pellet cellular debris. The virus-containing supernatant was subsequently filtered through a 45-µm filter and concentrated by centrifugation in 100 kDa Amicon tubes (Merck) for 30 min at 4,000×*g*. Virus titer was determined by TCID_50_ assay and calculated by the Reed–Muench method.

Following virus propagation, all variants were verified by whole genome sequencing. Briefly, virus isolates were prepared for whole genome sequencing using Nimagen EasySeq and sequenced using Illumina sequencing. Adapter and primer sequences were removed, and reads were mapped to the NC_045512.2 reference using Minimap and iVar (11, 12). Variants and pangolin lineages (13) were determined based on iVar consensus sequences with a minimum 80% base frequency; 70.5% of the B.1.351 sequence contained the nine lineage-specific mutations (aa:E:P71L, aa:N:T205I, aa:orf1a:K1655N, aa:S:D80A, aa:S:D215G, aa:S:K417N, aa:S:A701V, aa:S:N501Y, aa:S:E484K), and 29.5% of the sequence carried eight of the nine lineage-specific mutations and the Wuhan K417.

### Enzyme-Linked Immunosorbent Spot

Enumeration of specific IFN-γ and IL-4 secreting cells was performed by enzyme-linked immunosorbent spot (ELISpot) assay. For this assay, the monkey IFN-γ and IL-4 ELISpot kit from U-CyTech (Utrecht, The Netherlands) was used.

Freshly isolated peripheral blood mononuclear cells (PBMCs) were stimulated in triplicate with 1 µg/ml SARS-CoV-2 peptide pool covering the RBD of the spike protein aa 319–541 [PepMix SARS-CoV-2 (S-RBD), Cat no. PM-WCPV-S-RBD-2, JPT Innovative Peptide Solutions, Berlin, Germany]. The peptide pool contains 53 peptides of 15 aa, with an overlap of 11 aa. The negative control was medium alone, and the positive control was a PMA/ionomycin mixture (used at 50 ng/ml and 1 µg/ml, respectively). PBMCs (1.2 × 10^6^) were stimulated for 12–18 h in 300 µl RPMI-1640 medium supplemented with 10% fetal calf serum (FCS) and stimulus, in a 48-well tissue culture plate. After stimulation (37°C, 5% CO_2_), the non-adherent cells (approximately 50% from the start) were collected by three gentle washes using prewarmed RPMI-1640 medium supplemented with 10% FCS. After centrifugation, the cells were resuspended in 150 µl RPMI-1640 medium supplemented with 10% FCS and stimulus. For the enumeration of antigen-specific cytokine production, the 150-µl cell suspension was divided into three wells (approximately 2 × 10^5^ cells/well) of a Millipore MultiScreen 96-well filter plate. The filter plate was prewet for 1 min with 15 µl/well of 35% EtOH and then coated with anti-IFN-γ or anti-IL-4 mAb (1 h at 37°C or O/N at 4°C) and blocked with blocking buffer (1 h at 37°C or O/N at 4°C). The wells contained 50 µl medium with the same supplements as present during stimulation (including stimulus). After 5 h (IFN-γ) or overnight (IL-4) incubation, the cells were washed away, and before adding detector antibodies, an inactivation step with 300 µl 1% Triton X-100 for 1 h at RT was included. Spots were visualized using streptavidin-HRP and an AEC (3-amino-9-ethylcarbazole) coloring system. Spots were automatically counted using the A.EL.VIS reader from Sanquin (The Netherlands).

RBD-specific responses were calculated by subtracting the mean and 2 times the standard deviation of the number of spots measured in the medium control wells from the mean number of spots measured in the RBD peptide-stimulated wells and were expressed as spot-forming units (SFU) per million PBMCs.

### Statistical Analyses

Statistical significance of differences between the groups in RBD-binding IgG levels, neutralizing antibody titers, and viral sgRNA copies was calculated by using the Mann–Whitney test. A two-sided *α* level of 0.05 was used to determine significance. *P*-values <0.05 were considered significant.

## Results

### Immunogenicity

**Figure 1:**
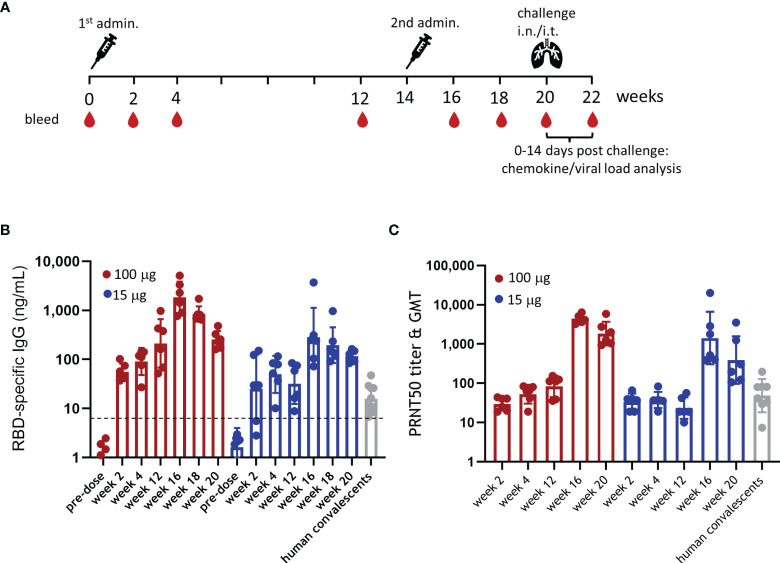
Experimental design and immunogenicity of ABNCoV2. **(A)** Schematic study design with non-human primates (NHP) (*N* = 6 per group) vaccinated intramuscularly with 100 or 15 μg ABNCoV2 in weeks 0 and 14 and challenged with severe acute respiratory syndrome coronavirus 2 (SARS-CoV-2) in week 20. Four (4) additional non-vaccinated NHP were added as controls. At regular intervals as indicated, animals were bled. **(B)** RBD-specific IgG was measured by ELISA, and **(C)** SARS-CoV-2 neutralizing antibodies were assessed by PRNT. For comparison, 10 human plasma samples positive for SARS-CoV-2 antibodies (human convalescents, gray symbols) were analyzed as well. Filled circles represent individual values and columns depict the geometric mean (ELISA, **B**) or geometric mean titers (PRNT, **C**) of the group +/geometric standard deviation. The horizontal dotted line in **(B)** represents the mean + 2 times the standard deviation of 50 untreated naive NHP to indicate background responses.

To evaluate the immunogenicity of ABNCoV2, six rhesus macaques per group were immunized intramuscularly with either 15 or 100 μg ABNCoV2 without adjuvant at week 0 and week 14 as outlined in **Figure 1A**. There were no local reactions observed in any animal at the vaccination site, i.e., the DRAIZE score was completely negative during 4 days post-immunizations (daily scoring). RBD-specific IgG by ELISA (**Figure 1B**), as well as SARS-CoV-2 neutralizing antibodies by live virus PRNT (**Figure 1C**), was already detected 2 weeks after the first immunization. Binding antibody levels increased with time in the high-dose group (100 μg ABNCoV2) from a geometric mean (GM) of 55 ng/ml at week 2 to GM 212 ng/ml by week 12; neutralizing antibodies increased from a PRNT50 geometric mean titer (GMT) of 30 to GMT 83 in the same period. In the low-dose group (15 μg ABNCoV2), antibodies induced after the first vaccination remained at a somewhat lower level (by week 12 reaching a significant difference of *p* < 0.05 compared with the high-dose group) with a GM of 25, 49, and 32 ng/ml in weeks 2, 4, and 12, respectively, and neutralizing antibodies at a GMT of 33, 37, and 23 at the same timepoints. Overall, binding as well as neutralizing antibodies induced by a single ABNCoV2 vaccination were durable within the monitored 3-month timeframe and were in the same range as those measured in 10 human convalescent samples (GM IgG 16 ng/ml and PRNT50 GMT 48), apart from higher levels (*p* = 0.0312) of RBD-binding antibodies in the 100-μg ABNCoV2 group. The second ABNCoV2 administration at week 14 significantly boosted both binding (*p* = 0.0087) (**Figure 1B**) and neutralizing (*p* = 0.0022) (**Figure 1C**) antibody responses in the two dose groups. At week 16, i.e., 2 weeks after the second ABNCoV2 administrations, IgG GM had increased to 1,835 ng/ml (high dose) and 284 ng/ml (low dose), i.e., 9-fold increase compared with 12 weeks after the first administration. Neutralizing antibodies had increased >50-fold to a GMT of 4,469 in the high-dose group and a GMT of 1,420 in the low-dose group. Although a decline in antibodies was detected in both vaccination groups by the time of challenge in week 20, the GM of binding antibodies was still 16 times higher in the high-dose group compared with human convalescent samples, and the GMT of neutralizing antibody titers was 38 times higher.

### Reduced Inflammatory Responses and Protective Efficacy Upon SARS-CoV-2 Challenge

**Figure 2:**
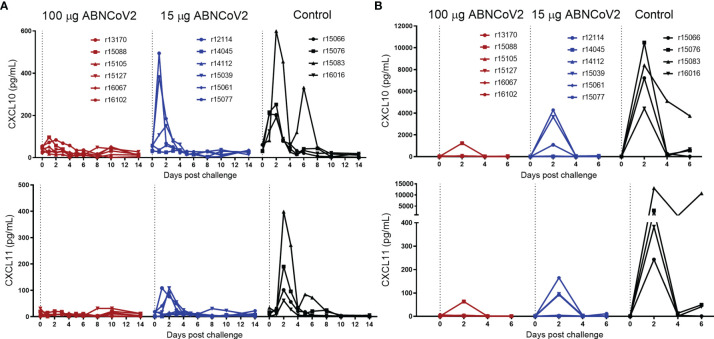
Chemokine changes following SARS-CoV-2 challenge. NHP (*N* = 6 per group) were vaccinated intramuscularly with 100 μg ABNCoV2 (red symbols) or 15 μg ABNCoV2 (blue symbols) in weeks 0 and 14 and challenged by the combined intranasal/intratracheal route with SARS-CoV-2 in week 20. As controls, four non-vaccinated rhesus macaques (black symbols) were challenged at the same time. CXCL10 (top graphs) and CXCL11 (bottom graphs) were measured in serum **(A)** and bronchoalveolar lavage (BAL) **(B)** on the days post-challenge as indicated. Each curve represents an individual animal. The symbol shapes identify individual animals as stated and the same symbols are used for the same animals throughout all post-challenge data.

To assess whether ABNCoV2 affords protection against infection, all 12 vaccinated animals and 4 non-vaccinated controls were challenged with 10^6^ TCID_50_ SARS-CoV-2 *via* the combined intranasal and intratracheal route at week 20, i.e., 6 weeks after the second vaccine administration. In the 2-week observation period post-challenge, only mild clinical symptoms were observed and there were no signs of enhanced respiratory disease (**Supplementary Table 2**). Indeed, cytokine profiling revealed a vaccine dose-dependent reduction of infection-induced inflammatory C-X-C motif chemokine ligand 10 (CXCL10) and 11 (CXCL11) in both serum (**Figure 2A**) and BAL (**Figure 2B**) compared with non-vaccinated controls. CXCL10, a chemokine described to contribute to exacerbating lung inflammation and progression to acute respiratory disease syndrome with importance as a prognostic and predictive marker for SARS-CoV-2 outcome (14), was transiently elevated in all four control NHP, with peak levels ranging from 191 to 599 pg/ml in serum and 4,423 to 10,473 pg/ml in BAL 2 days post-challenge. In the group vaccinated with 15 μg ABNCoV2, only three of the six animals showed transiently elevated levels of maximal 151–494 pg/ml CXCL10 in serum and 1,085–4,277 pg/ml in BAL. The high-vaccine dose group (100 μg ABNCoV2) showed no elevation of CXCL10 at any time post-challenge in serum, i.e., values were below 100 pg/ml, and only in a single animal 1,234 pg/ml was detected on post-challenge day 2 in BAL, a value well below those measured in control animals. Similarly, CXCL11, another chemokine responsible for the recruitment of inflammatory cells toward infected lungs (14), was transiently elevated in all control animals with peak mean amounts of 188 pg/ml in serum (**Figure 2A**) and 4,178 pg/mL in BAL (**Figure 2B**), compared with reduced peak mean amounts of 52 and 60 pg/ml in the low-vaccine dose group and 11 and 13 pg/ml in the high-vaccine dose group in serum and BAL, respectively. IL-1β and TNF-α (serum and BAL) as well as CCL5, CCL11, and IFN-γ (BAL) were not detected in any group, and changes of other cytokines measured were modest and/or more variable or restricted to the same control animal (**Supplementary Figures 1**, **2** showing serum and BAL, respectively).

**Figure 3:**
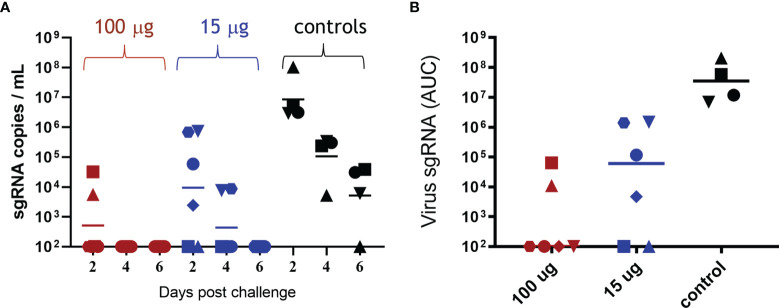
Viral load in bronchoalveolar lavage following SARS-CoV-2 challenge. Rhesus macaques were vaccinated and challenged as described in **Figure 2**. sgRNA was measured in bronchoalveolar lavage on days 2, 4, and 6 post-challenge. **(A)** sgRNA copies per ml BAL are shown for each animal in the 100-μg ABNCoV2 group by red symbols, in the 15-μg ABNCoV2 group by blue symbols, and in the non-vaccinated control group by black symbols. The symbol shapes identify individual animals; the same symbols are used for the same animals throughout all post-challenge data. Horizontal bars depict geometric mean values. **(B)** Total amount of sgRNA in BAL per animal (symbols) and median values (horizontal bars) are calculated as area under the curve (AUC). Significant differences by Mann–Whitney test were seen between each vaccinated group compared with the control group (*p* = 0.0095).

The efficacy of ABNCoV2 in terms of prevention of SARS-CoV-2 replication was monitored at regular intervals post-challenge. The level of sgRNA, attributed to replicating virus (10), was assessed by quantitative real-time PCR in BAL and throat and nose swabs. As depicted in **Figure 3A**, all four non-vaccinated control animals harbored a high number of sgRNA copies in BAL 2 days post-challenge (GM 8.5 × 10^6^, range 3 × 10^6^ to 1 × 10^8^ copies/ml), which decreased only slowly with time to a GM of 1 × 10^5^ copies/ml on post-challenge day 4 and 5 × 10^3^ copies/ml on post-challenge day 6, at which timepoint three of the four non-vaccinated controls were still positive with 6 × 10^3^ to 4 × 10^4^ sgRNA copies/ml. In contrast, no viral load in BAL was detected at any timepoint in the majority of animals (four of six) vaccinated with 100 μg ABNCoV2 (**Figure 3A**). Compared with controls, the only two positive animals in this group showed reduced levels of 6 × 10^3^ and 3 × 10^4^ sgRNA copies/ml on post-challenge day 2 (group GM of 511) and had completely cleared the virus 2 days later. The group vaccinated with the lower dose of 15 μg ABNCoV2 measured reduced GM of 9 × 10^3^ and 433 sgRNA copies/ml on post-challenge days 2 and 4, respectively, and total viral clearance on day 6. When calculating the area under the curve (AUC) to consider both level and kinetics of viral load (**Figure 3B**), a significant (*p* = 0.0095) difference of both vaccine dose groups could be shown compared with the control group. Similarly, viral load measured in throat swabs was significantly reduced in vaccinated animals (median AUC 1 × 10^3^ in the high-dose group and 2.25 × 10^4^ in the low-dose group), compared with controls (AUC 5.4 × 10^5^) in a vaccine dose-dependent manner (**Supplementary Figure 3A**). In nose swabs (**Supplementary Figure 3B**), though measuring a vaccine dose response, we failed to detect sgRNA in most control animals for unknown reasons and thereby also failed to show a significant difference such as those seen in BAL and throat.

**Figure 4:**
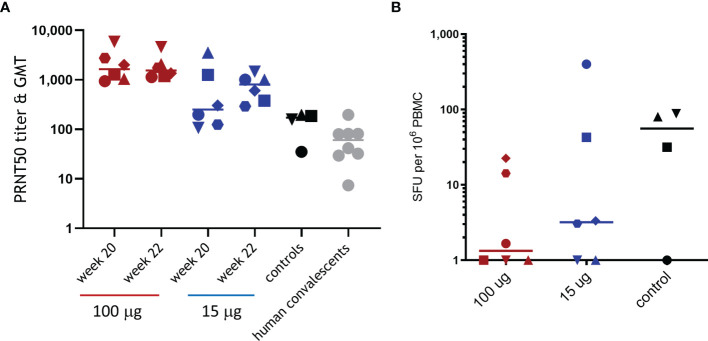
Immune responses following SARS-CoV-2 challenge. Rhesus macaques were vaccinated and challenged as described in **Figure 2**. SARS-CoV-2 neutralizing antibodies **(A)** were measured at the time of challenge (week 20) and 2 weeks post-challenge (week 22) by PRNT, and RBD-specific IFN-γ T-cell responses **(B)** were measured 2 weeks post-challenge by ELISpot. **(A)** Symbols represent individual PRNT50 titers; horizontal bars depict the GMT of the group. For comparison, PRNT50 titers and GMT of 10 human plasma samples positive for SARS-CoV-2 antibodies (human convalescents) are shown. **(B)** Symbols represent individual spot-forming units (SFU) per one million PBMCs; horizontal bars depict mean SFU per one million PBMCs. Different symbol shapes represent individual animals, and the same symbol shapes are used for the same animals throughout all post-challenge data.

Next, immune responses 2 weeks following challenge were measured to evaluate whether the significantly reduced viral load in the lungs and throat of ABNCoV2-vaccinated animals provided sufficient antigen for a booster response upon infection. All non-vaccinated control animals developed SARS-CoV-2 neutralizing antibodies (**Figure 4A**) and, apart from one animal, also RBD-specific IFN-γ T-cell responses with 32 to 88 SFU per million PBMCs (**Figure 4B**). Neutralizing antibodies in controls raised to a GMT of 119 2 weeks post-challenge (week 22) and were comparable to those measured in human convalescent samples (GMT 48). In contrast, high-dose ABNCoV2-vaccinated animals showed no increased neutralizing antibody titers 2 weeks post-challenge (week 22, GMT 1,764) compared with the time of challenge (week 20, GMT 1,854). Interestingly, T-cell responses were undetectable post-vaccination in this group and remained undetectable post-challenge in the majority (four of six) of these high-dose animals, while the remaining two animals recorded low T-cell responses (SFU/10^6^ PBMCs of 23 and 14) post-challenge. These findings are consistent with the largely undetectable viral load in this group. In line with a somewhat higher viral load post-challenge in the group vaccinated with 15 μg ABNCoV2, two animals with PRNT50 titers of 107 and 198, which were at the lower end of the group titer range at the time of challenge (PRNT50 of 107–3,539, GMT 390), experienced an increased PRNT50 titer of 1,462 and 1,006, respectively. One of these animals also recorded the highest RBD-specific IFN-γ T-cell response post-challenge (399 SFU/10^6^ PBMCs). No significant increase in neutralizing antibody titers was detected in the remaining four animals in that group. IL-4 T-cell responses were not detected in any animal in the study at any timepoint.

### Neutralization of SARS-CoV-2 Variants of Concern

**Figure 5:**
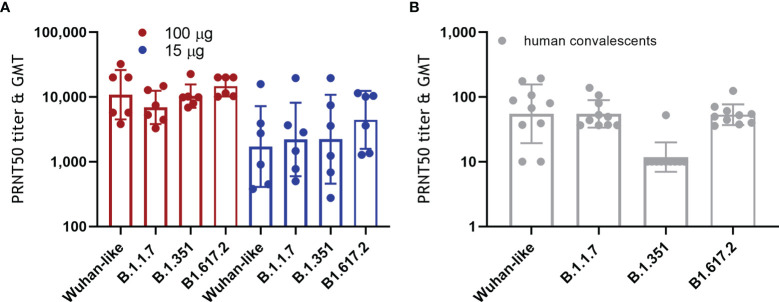
Neutralization of SARS-CoV-2 variants of concern. **(A)** NHP were vaccinated as described in **Figure 1**. Two weeks following the second administration, vaccine-induced antibodies were tested for their capacity to neutralize Wuhan-like SARS-CoV-2 (FR-4286) as well as the alpha (B.1.1.7), beta (B.1.351), and delta variants (B1.617.2) by PRNT. Filled circles represent PRNT50 titers of individual NHP, and columns depict the GMT of the group +/geometric standard deviation. Kruskal–Wallis test showed no statistically significant differences between the neutralization of Wuhan-like FR-4286 and the variants. **(B)** Ten human plasma samples positive for SARS-CoV-2 antibodies (human convalescents) were analyzed for comparison.

More and more SARS-CoV-2 variants of concern are emerging with increased resistance to antibody neutralization and reduced vaccine efficacy (2–4, 15, 16). We tested the capacity of NHP sera obtained 2 weeks after the second ABNCoV2 administration for their capacity to neutralize the Wuhan-like isolate (FR-4286) in comparison to the variants alpha (B.1.1.7, first identified in the UK), beta (B.1.351, first identified in South Africa), and delta (B1.617.2, first identified in India). As depicted in **Figure 5A**, no significant difference in neutralizing efficacy was measured between Wuhan-like FR-4286 (PRNT50 of 3,818–32,180, GMT 10,827), B.1.1.7 (PRNT50 of 3,282–14,713, GMT 6,899), B.1.351 (PRNT50 of 6,845–22,602, GMT 10,334), and B1.617.2 (PRNT50 of 10,092–20,000, GMT 14,544) in the 100-μg vaccine group. No significant effect on neutralization of variants was seen in the 15-μg vaccine group either (PRNT50 GMTs of 1,721, 2,207, 2,227, and 4,423 in the Wuhan-like FR-4286, B.1.1.7, B.1.351, and B1.617.2 specific assay, respectively), with the caveat of wide titer ranges in this group. In contrast, 10 human convalescent samples showed a significant drop in neutralizing titers against B.1.351 (GMT 12) compared with the Wuhan-like isolate (GMT 55), as reported by others (15) (**Figure 5B**).

## Discussion

The availability of safe and efficacious vaccines for global immunization against SARS-CoV-2 including emerging variants of concern is of utmost importance to fight the current pandemic. Our data in the rhesus macaque model demonstrated that two administrations of the non-adjuvanted cVLP-based vaccine ABNCoV2, which displays the RBD of the spike protein of SARS-CoV-2, induced robust vaccine dose-dependent neutralizing antibody responses that peaked at 29-fold and 92-fold higher GMT in the 15- and the 100-μg dose groups, respectively, compared with human convalescent samples. The persistence of antibody responses measured throughout the 3 months between the two vaccine administrations may indicate long-lived plasma cells, such as those seen for other VLP vaccines (6, 17). Complete protection from viral load was measured in BAL in four out of six NHP in the 100-μg ABNCoV2 group and in two out of six NHP in the 15-μg dose group, with reduced levels compared with non-vaccinated controls in the remaining animals. Moreover, the levels of infection-induced CXCL10 and CXCL11 were reduced. Both cytokines are responsible for the recruitment of inflammatory cells toward infected lungs and have been associated with intra-alveolar hemorrhage in COVID-19 patients (14, 18, 19). These data are in line with NHP studies of licensed vaccines that have already proven efficacious in humans: mRNA-1273 induced 15 times the neutralizing antibody GMT of convalescent samples and total viral clearance in BAL in seven out of eight NHP post-challenge (20), while mRNA BNT162b2 at the clinical dose of 30 μg yielded 10 times the neutralizing antibody levels of convalescent samples, and at 100 μg (>3 times the clinical dose), it led to complete viral clearance in BAL (21), whereby not all non-vaccinated controls showed viral load, potentially indicating a less severe challenge. Neutralizing antibodies elicited by ChAdOx1 nCoV-19 were not compared with convalescent samples, but a priming vaccination with the vaccine was reported to result in titers similar to those obtained in SARS-CoV-2-infected NHP (22), which was also the case after a single ABNCoV2 vaccination. A prime-boost ChAdOx1 nCoV-19 vaccination resulted in protection from viral load in BAL in four out of six animals (22). Another adenoviral vaccine, Ad26-S.PP, induced 4-fold higher median neutralizing antibody titers compared with convalescent NHP and humans and total viral clearance in BAL after challenge (23). Similarly, viral load in the respiratory tract (nasal or throat swabs) was generally reduced in vaccinated NHP compared with controls in these studies, though no significance could be shown for BNT162b2 or ChAdOx1 nCoV-19 (21, 22). Throat swabs analyzed in the current study demonstrated significantly lower viral load compared with controls even in the low 15-μg ABNCoV2 dose group, potentially indicating the inhibition of transmission, with the caveat that we were unable to detect significant differences with controls in either dose group in terms of viral load in nose swabs. This was due to the unexpected inability to detect viral sgRNA in nose swabs in three of the four control animals. Since ABNCoV2 induced comparable neutralizing antibodies and protection in the rhesus macaque model as licensed vaccines in the same model, it is tempting to speculate that ABNCoV2 may also be efficacious against SARS-CoV-2 in humans, in which the licensed vaccines have demonstrated protection against symptomatic COVID-19 of up to 95% (24). Although a single causal correlate of protection has not been identified, neutralizing antibodies have been shown to correlate with disease outcome in macaques (23) and their levels are highly predictive of protection in humans (25–28). For this reason, the high (92-fold above the GMT in convalescents) peak neutralizing antibody titer induced by two administrations of 100 μg ABNCoV2 is very encouraging. Khoury and colleagues estimated the neutralization level for 50% protection against detectable SARS-CoV-2 infection to be about 20% of the mean convalescent level (25). Of course, the neutralizing antibody titers shown here were generated in NHP, and responses in humans induced by ABNCoV2 remain to be demonstrated in currently ongoing clinical trials.

It is remarkable that we observed no significant differences when we compared SARS-CoV-2 alpha, beta, or delta neutralizing titers to those measured against an original isolate, while reduced neutralizing antibody levels against variants of concern, especially against beta, have been reported for other vaccines (2, 15, 16, 29–32), in some instances >10-fold lower levels (33–35). The obvious species difference seems unlikely to account for this, since serum from mice, hamsters, and rhesus macaques vaccinated with a chimpanzee adenovirus-based SARS-CoV-2 vaccine consistently exhibited reduced neutralizing activity against viruses containing the E484K mutation present in the beta variant (29). Instead, one may speculate that the greater cross-neutralization may be due to a focus of ABNCoV2-induced antibodies to its displayed RBD, the main target for neutralizing antibodies, rather than the full spike protein that is used by the current licensed platforms. In line with the predicted translation of reduced levels of neutralizing antibodies to reduced efficacy (25), mRNA BNT162b2 that had demonstrated 95% efficacy in phase 3 (24) showed 90% effectiveness against documented infection with the B.1.1.7 variant and 75% against B.1.351 in a mass immunization campaign in Qatar (36). Ad26-S.PP demonstrated a difference in the efficacy from 74% against moderate to severe disease in the United States, which was dominated by the Wuhan isolate, to an efficacy of 52% in B.1.351 variant dominated South Africa (37), while the efficacy of ChAdOx1 nCoV-19 was reported as low as 10% against mild to moderate disease induced by the B.1.351 variant in a trial performed in South Africa (2). Vaccine efficacy against severe disease, which is reported to be less effected for other vaccines, was undetermined in this trial.

In light of the increasing prevalence of variants of concern and the emergence of new variants, the induction of comparable Wuhan (FR-4286), alpha, beta, and delta neutralizing antibody titers in ABNCoV2-vaccinated NHP shown here is highly promising.

In summary, ABNCoV2 induced strong and long-lasting neutralizing antibody responses and efficacy against SARS-CoV-2 in NHP. Together with the comparable variant neutralization capacity seen in the serum of vaccinated animals, these data may indicate a potential for broad protection afforded by the ABNCoV2 vaccine, which is currently in clinical testing.

## Data Availability Statement

The original contributions presented in the study are publicly available. These data can be found here: NCBI GenBank accession nos. MZ314997 and OM444216 and GISAID accession nos. EPI_ISL_852748 and EPI_ISL_678615.

## Ethics Statement

The animal study was reviewed and approved by the Dutch “Centrale Commissie Dierproeven” (AVD5020020209404-2) according to Dutch law, article 10a of the “Wet op de Dierproeven,” and BPRC’s Animal Welfare Body (IvD).

## Author Contributions

AV designed and monitored the study, analyzed the data, and wrote the manuscript. MI propagated the Wuhan-like SARS-CoV-2 isolate (Freiburg, FR-4286) and variants of concern, performed the live virus PRNT experiments, and analyzed the data. SP supervised the PRNT experimental design, supplied the variants of concern, and reviewed the data. MI and SP contributed to the editing of the manuscript. EV analyzed the data. BV performed the experiments, analyzed the data, and coordinated the study post-infection. WB reviewed the data. GK took part in the study design, performed the experiments, reviewed and analyzed the data, coordinated the study, and contributed to the editing of the manuscript. PM took part in the study design, performed the experiments, reviewed and analyzed the data, coordinated the study, and contributed to the editing of the manuscript. MN and AS supplied the vaccine and edited the manuscript. CS analyzed the data. HH participated in the conception of the study and reviewed the content of the manuscript. PC took part in the study design. SV performed full genome sequencing of the SARS-CoV-2 variants and subsequent bioinformatics analysis of the sequences. All authors contributed to the article and approved the submitted version.

## Funding

Production of the ABNCoV2 vaccine was supported by a European Commission H2020 grant (Grant agreement ID: 101003608). SARS-CoV-2 variant neutralization assay work was supported by the Carlsberg Foundation (Semper Ardens) and the Independent Research Fund Denmark (0214-00001B).

## Conflict of Interest

AS are listed as co-inventors on a patent application covering the AP205 CLP vaccine platform technology (WO2016112921 A1). AV, CS, HH, and PC are employees of Bavarian Nordic, a vaccine development company that has licensed ABNCoV2 for further development and commercialization. MN and AS were employed by AdaptVac Aps.

The remaining authors declare that the research was conducted in the absence of any commercial or financial relationships that could be construed as a potential conflict of interest.

## Publisher’s Note

All claims expressed in this article are solely those of the authors and do not necessarily represent those of their affiliated organizations, or those of the publisher, the editors and the reviewers. Any product that may be evaluated in this article, or claim that may be made by its manufacturer, is not guaranteed or endorsed by the publisher.
