# Supplementary material for: A Capsid Virus-Like Particle-Based SARS-CoV-2 Vaccine Induces High Levels of Antibodies and Protects Rhesus Macaques
Source: Front Immunol. 2022 Apr 5;13:857440. doi: 10.3389/fimmu.2022.857440 (PMC9037084; doi:10.3389/fimmu.2022.857440)
Supplement: Supplementary file 1 [file DataSheet_1.docx]

Supplementary Material

## Supplementary Figures


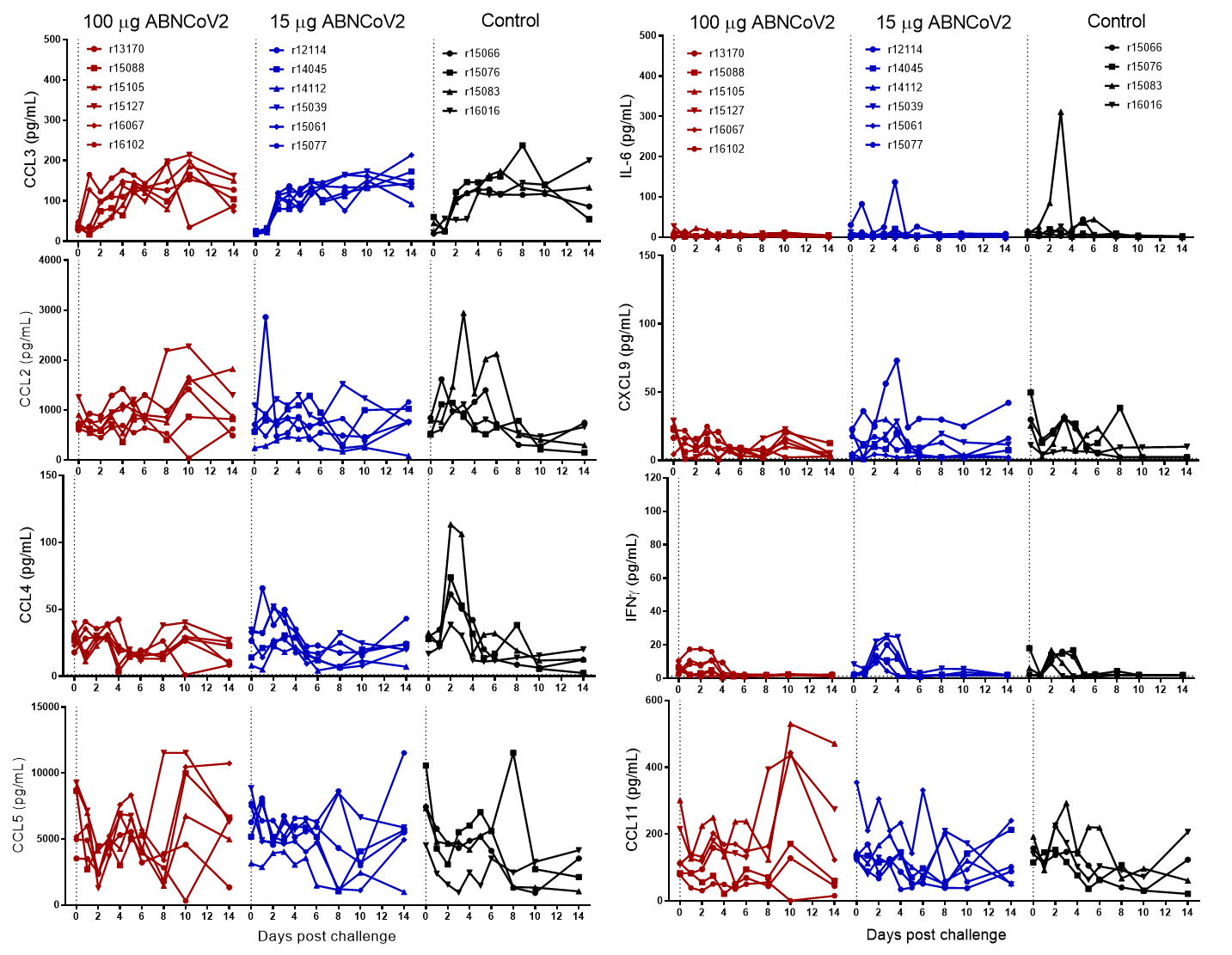


**Supplementary Figure 1.** Chemokine changes in serum following SARS-CoV-2 challenge.

Different chemokines and cytokines as indicated were measured in serum on the day of challenge and every second day thereafter.


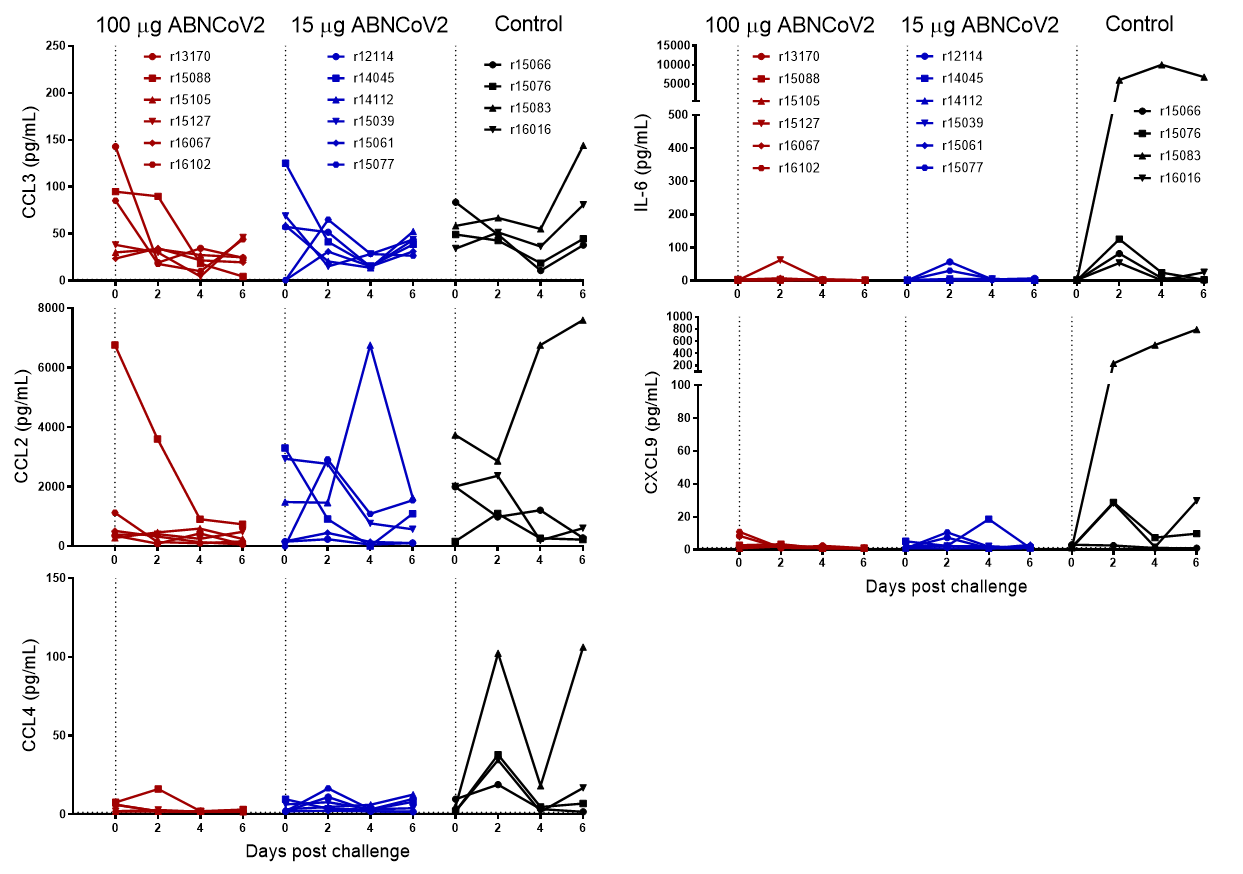


**Supplementary Figure 2.** Chemokine changes in BAL following SARS-CoV-2 challenge.

Different chemokines and cytokines as indicated were measured in BAL on the day of challenge and on days 2, 4 and 6 post challenge.


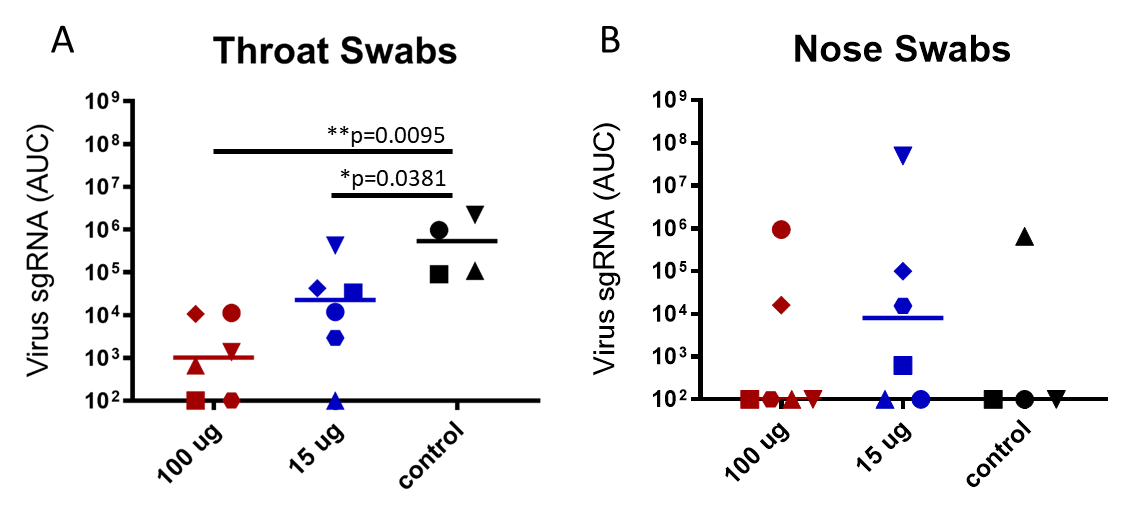


**Supplementary Figure 3.** Viral load in throat and nose swabs following SARS-CoV-2 challenge.

NHP (N=6 per group) were vaccinated intramuscularly with 100 μg ABNCoV2 (red symbols) or 15 μg ABNCoV2 (blue symbols) in week 0 and 14 and challenged by the combined intranasal/intratracheal route with SARS-CoV-2 in week 20. As controls, 4 non-vaccinated rhesus macaques (black symbols) were challenged at the same time.
SgRNA was assessed in throat swabs (A) and nose swabs (B). Total amount of sgRNA per animal (symbols) and median values (horizontal bars) are calculated as area under the curve (AUC). The symbol shapes identify individual animals; the same symbols are used for the same animals throughout all post-challenge data. Significant differences by Mann-Whitney test with the respective p values are indicated.

## Supplementary Tables

| **Group** | **Animal ID** | **Age** | **Weight (kg)** | **Sex** |
| --- | --- | --- | --- | --- |
| **ABNCoV2**  **100 μg** | R13170 | 7 | 11.7 | Female |
|  | R15088 | 5 | 6.05 | Female |
|  | R15105 | 5 | 7.6 | Male |
|  | R15127 | 5 | 9.1 | Male |
|  | R16067 | 4 | 7.8 | Male |
|  | R16102 | 4 | 7.2 | Male |
| **ABNCoV2**  **15 μg** | R15039 | 5 | 5.45 | Female |
|  | R15061 | 5 | 5.85 | Female |
|  | R14112 | 6 | 11.1 | Male |
|  | R15077 | 5 | 7.1 | Male |
|  | R12114 | 8 | 9.6 | Male |
|  | R14045 | 6 | 9.55 | Male |
| **Control** | R15076 | 5 | 8.1 | Male |
|  | R16016 | 4 | 7.25 | Male |
|  | R15066 | 5 | 9.8 | Male |
|  | R15083 | 5 | 9.95 | Male |

**Supplementary Table 1.** Group allocation at study enrolment.

| **Group** | **Animal** | **post challenge day** | | | | | | | | | | | | | | |
| --- | --- | --- | --- | --- | --- | --- | --- | --- | --- | --- | --- | --- | --- | --- | --- | --- |
|  |  | **0** | **1** | **2** | **3** | **4** | **5** | **6** | **7** | **8** | **9** | **10** | **11** | **12** | **13** | **14** |
| **100 μg**  **ABNCoV2** | **R13170** | 0.0 | 0.5 | 1.5 | 1.0 | 0.5 | 1.0 | 1.0 | 2.0 | 1.0 | 0.5 | 1.0 | 0.0 | 0.0 | 0.0 | 0.5 |
|  | **R15088** | 0.0 | 0.5 | 1.5 | 1.0 | 0.5 | 2.0 | 0.0 | 1.0 | 0.5 | 0.5 | 0.0 | 0.0 | 0.0 | 0.0 | 0.5 |
|  | **R15105** | 0.5 | 1.0 | 1.0 | 0.0 | 2.0 | 1.0 | 2.5 | 2.0 | 3.0 | 2.5 | 0.0 | 1.0 | 2.0 | 2.0 | 2.0 |
|  | **R15127** | 0.5 | 1.5 | 2.0 | 1.0 | 1.0 | 2.0 | 2.5 | 2.0 | 1.0 | 1.5 | 0.0 | 0.5 | 0.0 | 0.0 | 0.0 |
|  | **R16067** | 0.5 | 0.5 | 2.0 | 0.5 | 2.0 | 0.0 | 3.5 | 1.5 | 2.0 | 0.0 | 1.0 | 0.0 | 0.0 | 2.0 | 1.0 |
|  | **R16102** | 0.5 | 1.5 | 3.0 | 1.5 | 1.0 | 2.0 | 3.5 | 2.5 | 3.0 | 2.0 | 1.0 | 0.0 | 0.0 | 2.0 | 1.0 |
|  |  |  |  |  |  |  |  |  |  |  |  |  |  |  |  |  |
| **15 μg**  **ABNCoV2** | **R12114** | 0.0 | 0.5 | 1.0 | 0.0 | 0.5 | 1.0 | 1.0 | 1.0 | 1.5 | 0.0 | 0.0 | 0.0 | 0.0 | 0.5 | 0.0 |
|  | **R14045** | 0.0 | 0.5 | 1.0 | 0.0 | 1.5 | 2.0 | 4.0 | 2.0 | 1.5 | 2.0 | 1.0 | 2.0 | 2.0 | 2.5 | 0.0 |
|  | **R14112** | 0.0 | 0.0 | 0.0 | 0.0 | 3.0 | 1.5 | 2.0 | 0.5 | 0.5 | 0.5 | 0.5 | 1.0 | 0.0 | 0.5 | 0.5 |
|  | **R15039** | 0.0 | 0.5 | 3.5 | 2.0 | 2.0 | 0.0 | 0.0 | 4.0 | 1.0 | 0.5 | 1.0 | 0.0 | 0.0 | 0.0 | 0.5 |
|  | **R15061** | 0.0 | 0.5 | 1.5 | 1.0 | 2.0 | 1.0 | 1.0 | 2.0 | 1.0 | 0.5 | 0.0 | 0.0 | 0.0 | 0.0 | 0.5 |
|  | **R15077** | 0.0 | 0.0 | 0.0 | 1.0 | 3.0 | 5.5 | 1.5 | 1.5 | 0.5 | 4.5 | 1.5 | 0.0 | 0.0 | 0.0 | 0.5 |
|  |  |  |  |  |  |  |  |  |  |  |  |  |  |  |  |  |
| **control** | **R15066** | 0.0 | 1.0 | 1.0 | 0.5 | 0.5 | 0.0 | 2.0 | 0.5 | 1.0 | 0.0 | 0.0 | 0.0 | 0.0 | 1.0 | 0.0 |
|  | **R15076** | 0.0 | 0.5 | 0.0 | 1.5 | 3.5 | 0.5 | 1.0 | 2.0 | 1.0 | 2.0 | 1.5 | 0.0 | 0.0 | 0.0 | 0.5 |
|  | **R15083** | 0.0 | 0.0 | 1.0 | 5.5 | 4.0 | 1.0 | 4.5 | 3.5 | 3.0 | 1.0 | 1.0 | 0.0 | 0.0 | 1.0 | 0.0 |
|  | **R16016** | 0.0 | 1.0 | 0.0 | 1.5 | 0.5 | 0.5 | 1.0 | 1.0 | 1.0 | 2.5 | 1.5 | 0.0 | 0.0 | 0.0 | 0.5 |

**Supplementary Table 2.** Clinical scores following SARS-CoV-2 challenge.

A scoring system was used including clinical appearance, respiratory rate, recumbency, responsiveness, fever and weight loss with individual category scores of up to 10. The highest daily sum of morning or afternoon scores for an animal determined the severity of disease. Clinical disease severity was classified as mild (0-12), moderate (13-20), severe (>21). Animals would have been euthanized immediately if they reached an individual category score of 10 or a total score of ≥21. However, only mild symptoms were observed with total scores well below 10. Total daily scores (the mean of the morning and afternoon scores) are shown on the day of challenge (post challenge day 0) and on 14 consecutive days post challenge.
